# Supplementary material for: Opposing effects of T cell receptor signal strength on CD4 T cells responding to acute versus chronic viral infection
Source: eLife. 2021 Mar 8;10:e61869. doi: 10.7554/eLife.61869 (PMC7943189; doi:10.7554/eLife.61869)
Supplement: Supplementary file 1. [file elife-61869-supp1.docx]

| **APL** | | **EC50 [M]** |  | **APL** | | **EC50 [M]** |
| --- | --- | --- | --- | --- | --- | --- |
| **GP61wt** | | 5.2E-09 |  | **V71** | **W** | 5.9E-08 |
| **Y68** | **A** | 1.3E-06 |  | **V71** | **P** | 2.2E-06 |
| **Y68** | **V** | 7.2E-08 |  | **V71** | **M** | 1.2E-08 |
| **Y68** | **L** | 6.5E-06 |  | **V71** | **C** | 2.1E-07 |
| **Y68** | **I** | 1.3E-07 |  | **Y72** | **S** | 4.2E-05 |
| **Y68** | **S** | 2.6E-06 |  | **Y72** | **T** | 1.7E-05 |
| **Y68** | **T** | 4.8E-07 |  | **Y72** | **E** | 2.1E-07 |
| **Y68** | **N** | 1.6E-07 |  | **Y72** | **R** | 2.1E-06 |
| **Y68** | **E** | 7.0E-05 |  | **Y72** | **F** | 9.6E-07 |
| **Y68** | **Q** | 6.7E-06 |  | **Y72** | **P** | 6.3E-08 |
| **Y68** | **K** | 1.1E-06 |  | **Y72** | **M** | 5.6E-05 |
| **Y68** | **R** | 1.5E-06 |  | **Q73** | **L** | 8.7E-08 |
| **Y68** | **H** | 3.1E-07 |  | **Q73** | **S** | 2.5E-05 |
| **Y68** | **W** | 1.4E-08 |  | **Q73** | **D** | 3.2E-06 |
| **Y68** | **P** | 1.1E-05 |  | **Q73** | **E** | 3.1E-07 |
| **Y68** | **C** | 1.0E-06 |  | **Q73** | **K** | 8.3E-06 |
| **Y68** | **M** | 1.6E-07 |  | **Q73** | **W** | 2.6E-07 |
| **K69** | **G** | 5.2E-06 |  | **Q73** | **F** | 1.1E-07 |
| **K69** | **A** | 3.2E-08 |  | **Q73** | **C** | 2.9E-07 |
| **K69** | **V** | 5.0E-07 |  | **F74** | **V** | 8.7E-07 |
| **K69** | **L** | 5.5E-07 |  | **F74** | **I** | 1.9E-05 |
| **K69** | **S** | 1.2E-07 |  | **F74** | **Y** | 1.3E-06 |
| **K69** | **T** | 8.1E-08 |  | **K75** | **R** | 5.1E-07 |
| **G70** | **V** | 3.5E-07 |  | **S76** | **D** | 1.0E-06 |
| **G70** | **L** | 1.2E-07 |  | **S76** | **E** | 5.7E-07 |
| **G70** | **I** | 3.5E-06 |  | **S76** | **Q** | 5.9E-08 |
| **G70** | **K** | 5.8E-08 |  | **S76** | **K** | 2.0E-07 |
| **G70** | **R** | 1.1E-07 |  | **S76** | **R** | 2.2E-06 |
| **G70** | **F** | 1.4E-05 |  | **S76** | **W** | 7.9E-05 |
| **G70** | **Y** | 1.7E-07 |  | **S76** | **F** | 4.5E-07 |
| **G70** | **M** | 6.2E-08 |  | **S76** | **Y** | 5.3E-06 |
| **V71** | **G** | 9.3E-07 |  | **S76** | **P** | 3.8E-07 |
| **V71** | **S** | 1.4E-07 |  | **S76** | **C** | 5.9E-08 |
| **V71** | **D** | 1.4E-06 |  | **V77** | **G** | 1.8E-07 |
| **V71** | **N** | 8.4E-06 |  | **V77** | **S** | 3.6E-08 |
| **V71** | **E** | 9.5E-06 |  | **V77** | **D** | 4.8E-07 |
| **V71** | **Q** | 2.9E-06 |  | **V77** | **E** | 6.5E-07 |
| **V71** | **H** | 2.2E-08 |  | **V77** | **P** | 4.0E-08 |
